# Pre-sowing Seed Treatment with 24-Epibrassinolide Ameliorates Pesticide Stress in *Brassica juncea* L. through the Modulation of Stress Markers

**DOI:** 10.3389/fpls.2016.01569

**Published:** 2016-11-02

**Authors:** Anket Sharma, Sharad Thakur, Vinod Kumar, Mukesh K. Kanwar, Anup K. Kesavan, Ashwani K. Thukral, Renu Bhardwaj, Pravej Alam, Parvaiz Ahmad

**Affiliations:** ^1^Plant Stress Physiology Lab, Department of Botanical and Environmental Sciences, Guru Nanak Dev UniversityAmritsar, India; ^2^Department of Molecular Biology and Biochemistry, Guru Nanak Dev UniversityAmritsar, India; ^3^Department of Botany and Environmental Science, Sri Guru Granth Sahib World UniversityFatehgarh Sahib, India; ^4^Biology Department, College of Science and Humanities, Prince Sattam bin Abdulaziz UniversityAlkharj, Saudi Arabia; ^5^Department of Botany and Microbiology, Faculty of Science, King Saud UniversityRiyadh, Saudi Arabia; ^6^Department of Botany, S. P. CollegeSrinagar, India

**Keywords:** mustard, brassinosteroids, pigments, total phenols, phenylalanine ammonialyase, GC-MS, HPLC

## Abstract

The present experiment was designed to assess the effects of seed soaking with 24-epibrassinolide (EBR) on the physiology of *Brassica juncea* L. seedlings grown under imidacloprid (IMI) toxicity. Application of EBR increased the length of seedlings, dry weight, and pigment contents, polyphenols, total phenols, and organic acids under IMI toxicity. The expression of genes coding key enzymes of pigment, phenols, polyphenols, and organic acid biosynthetic pathways was also studied including *CHLASE* (chlorophyllase), *PSY* (phytoene synthase), *CHS* (chalcone synthase) and *PAL* (phenylalanine ammonialyase), *CS* (citrate synthase), *SUCLG1* (succinyl Co-A ligase,), *SDH* (succinate dehydrogenase), *FH* (fumarate hydratase), *MS* (malate synthase). Multiple linear regression (MLR) analysis revealed that IMI application regressed negatively on seedling length, dry weight and total chlorophyll content. However, EBR seed treatment regressed positively on all the parameters studied. Moreover, interaction between IMI and EBR showed positive regression for growth parameters, content of pigments, total polyphenol, total phenol and malate, and expression of *PSY* and *PAL*. Negative interactions were noticed for the contents of fumarate, succinate and citrate, and expression of *CHS* and all genes studied related to organic acid metabolism. In conclusion, EBR enhanced the growth and contents of all studied metabolites by regulating the gene expression of *B. juncea* seedlings under IMI stress.

## Introduction

*Brassica juncea* L. is an important oil yielding as well as vegetable crop. Various insect pests including termites, aphids, leafhoppers, and other sucking insects infest it. Pesticides are widely utilized to control insect pests, and imidacloprid (IMI) being the most preferred pesticide to control these soil and sap-sucking insects (El-Naggar and Zidan, 2013; Ko et al., 2014). To protect pesticidal air pollution and protect plants from soil insects, IMI is applied to soil before seed sowing (Bonmatin et al., 2005). However, pesticide application also causes phytotoxicity to plants resulting in their impaired growth and chlorophyll degradation (Sharma et al., 2015; Singh et al., 2016). Moreover, in plants under pesticide stress, secondary metabolites like phenolic compounds (Sharma et al., 2016a), carotenoids, anthocyanins, xanthophylls (Tan et al., 2012; Kilic et al., 2015; Sharma et al., 2016b), and organic acids (Ding et al., 2014) were reported to enhance.

Brassinosteroids (BRs) are plant steroids, which are well-known to increase resistance in plants against various abiotic stresses like heavy metals and pesticides (Hayat et al., 2010; Sharma et al., 2012, 2013, 2015). 24-epibrassinolide (EBR) has similar biological functions to those of original form of brassinolide and is mostly used in physiological studies (Vardhini and Anjum, 2015). Exogenous application of EBR in crop plants has been reported to enhance their growth, pigment contents, photosynthetic efficiency, enzymatic, and non-enzymatic antioxidants (Xia et al., 2006; Sharma et al., 2012, 2013, 2015, 2016a; Vardhini and Anjum, 2015; Zhou et al., 2015). It has also been reported that exogenous application of EBR to plants decreases the pesticide residues (Xia et al., 2009; Zhou et al., 2015; Sharma et al., 2016c,d). Phenolic compounds and pigments which act as antioxidants including anthocyanins, carotenoids, and xanthophylls also got enhanced after the exogenous application of EBR in different plants (Chen et al., 2011; Nakabayashi et al., 2014; Sharma et al., 2016a,d). Moreover, EBR application may also modulates gene expression in plants to enhance their resistance against pesticide stress (Xia et al., 2009; Sharma et al., 2015; Zhou et al., 2015). Treatment of EBR via seed soaking before sowing has been reported to ameliorate the pesticide toxicity in plants (Sharma et al., 2012, 2016a). Keeping in mind the protective roles of EBRs in plants under pesticide toxicity, the present study was undertaken to assess the effects of EBR seed soaking before sowing on *B. juncea* seedlings grown under IMI stress.

## Materials and methods

### Raising of plant material

Seeds of *B. juncea* (cv. RLC-1) were given pre-sowing treatment with 24-epibrassinolide (EBR) solutions (0 and 100 nM EBR/L) for 8 h. Petri-plates were lined with Whatman1 filter paper and were supplemented with different imidacloprid (IMI) concentrations (0, 150, 200, and 250 mg IMI/L). The EBR treated seeds were rinsed with distilled water and grown in Petri-plates supplemented with IMI solutions (three petri-plates for each treatment). The Petri-plates were kept in seed germinator (temperature = 25 ± 0.5°C, photoperiod = 16 h, light intensity = 175 μmol m^−2^ s^−1^) and the seedlings were harvested 10 days after sowing for further analysis.

### Estimation of growth parameters

Length of seedling was measured by scale and for dry weight, the seedlings were weighed after drying them at 70°C for 48 h.

### Estimation of pigment content (chlorophyll, carotenoid, anthocyanin, and xanthophyll content)

Chlorophyll contents were estimated according to the method given by Arnon (1949), whereas carotenoid content was estimated as described by Kapoor et al. (2014). One gram of fresh seedlings were crushed in 4 ml of 80% acetone. The extract was then centrifuged (4°C) at 12,000 × rpm for 20 min. The supernatant was used to determine the contents of chlorophylls and carotenoids. Absorbance was taken using spectrophotometer at 645 and 663 nm for chlorophylls, whereas 480 and 510 nm for carotenoids.

Method given by Mancinelli (1984) was followed to determine the anthocyanin content. One gram of fresh seedlings were crushed in 3 ml of extraction solvent containing 0.03 ml of hydrochloric acid (HCl), 0.6 ml of distilled water, and 2.37 ml of methanol. The crushed sample was then centrifuged (4°C) at 12,000 × rpm for 20 min. and the absorbance was recorded spectrophotometerically at 530 and 657 nm.

Association of Official Agricultural Chemists (AOAC) procedure given by Lawrence (1990) was followed to determine the xanthophyll content. Fifteen milliliter of solvent mixture (5 ml hexane, 3.5 ml acetone, 3 ml ethanol, 3.5 ml toluene) was added to 50 ml flask containing 25 mg dried seedling powder. The flask was shaken for 10–15 min. After shaking, 1 ml of 40% methanolic potassium hydroxide (KOH) was poured into the flask and was incubated at 56°C for 20 min (water bath) followed by another incubation in dark for 1 h. After incubation, 15 ml of hexane was added to the flask and then shaken well for 1 min. The 50 ml volume of flask was made up by adding 10% sodium sulfate solution followed by incubation in dark for 1 h. The upper phase was collected in 25 ml volumetric flask and the makeup of the volume was done using hexane. Absorbance was taken using spectrophotometer at 474 nm.

### Determination of total phenols

Method given by Singleton and Rossi (1965) was followed to determine the total phenol content. One gram of fresh seedlings were homogenized in 5 ml of 60% ethanol followed by incubation at 60°C for 30 min. One hundred and twenty five microliter sample from incubated mixture was added to 0.625 ml of Folin-Ciocalteu (FC) reagent and 0.5 ml of 7.5% Na_2_CO_3_ (sodium carbonate) followed by incubation for 2 h at 25°C. Absorbance was taken at 765 nm by spectrophotometer.

### Quantification of polyphenols using ultra high performance liquid chromatography (UHPLC)

Method described by Sharma et al. (2016a) was followed to determine the polyphenol content. One gram of fresh seedlings were crushed in 5 ml of 80% methanol followed by centrifugation at 12,000 × rpm for 15 min. Ten microliter of sample was injected into UHPLC system equipped with SPD-M20A photodiode array detector. Analytical column used was C_18_ (column length = 150 mm; internal diameter = 4.6 mm; pore size = 100 Å; company = Spincotech) and wavelength selected for absorbance was 280 nm. Acetic acid (0.01%) and methanol (HPLC grade, 100%) were used as mobile phase A and B, respectively. Flow rate was set at 1 ml/min. Gradient information: 0–1 min, 30% B; 12 min, 45% B; 15 min, 75% B; 16.6 min, 50%; 20 min, 25%; 21 min, 30%. Program was terminated at 22 min (elution time-4 min). The polyphenols were identified and quantified using standards which were analyzed before running plant samples (Shimadzu LabSolutions software).

### Quantification of organic acids using gas chromatography-mass spectrometry (GC-MS)

#### Sample preparation

Method given by Chen et al. (2001) was modified to estimate organic acids using GC-MS. Extraction of organic acids was done by adding 0.5 ml of 0.5 N HCl and 0.5 ml of methanol to 50 mg of dried seedling powder followed by shaking for 3 h and then centrifuged at 12,000 × rpm for 10 min. To the supernatant, 300 μl of methanol and 100 μl of 50% sulfuric acid (H_2_SO_4_) were added followed by overnight incubation in water bath at 60°C. The mixture was cooled down to 25°C and 800 μl of chloroform and 400 μl of distilled water were added to it followed by vortexing for 1 min. The lower chloroform layer was used to estimate organic acids using GC-MS.

#### Analysis using GC-MS

To estimate organic acid content, 2 μl of sample (lower chloroform layer) was injected into GC-MS system. GC conditions: Carrier gas used was helium, initial column temperature was 50°C (hold for 1 min) which was increased at 25°C/min to 125°C followed by further increase at 10°C/min to 300°C and held for 15 min. Injection temperature was 250°C, mode of injection was split, flow of gas in column was 1.7 ml/min, and analytical column used was DB-5ms. MS conditions: Ion source temperature was set at 200°C and interface temperature was 280°C, solvent cut time was 3 min, detector gain mode: relative. Organic acids *viz*. citrate, fumarate, malate, and succinate were detected by comparing their mass spectra using National Institute of Standard and Technology (NIST08s) and Wiley 7 library. The quantification of these organic acids was done using standard curve.

### Gene expression through quantitative real time PCR (qRT-PCR)

Trizol method (Invitrogen) was followed to extract total RNA from 100 mg of *B. juncea* seedlings. RNA to cDNA kit (Invitrogen) was used to make cDNA from 1 μg of total RNA. No DNase was used in the experiment and to make sure that there was no genomic DNA contamination, a PCR reaction was set using total RNA extracted as template and no amplified product was detected. EMBL and Genbank were used to design gene specific primers (Table 1) and *actin* was taken as an internal control. qRT-PCR was done in three biological replicates using Power SYBR green PCR master mix and StepOne qRT-PCR system (Applied Biosystems). PCR conditions: initial denaturation at 95°C for 10 min; 40 cycles of denaturation at 95°C (15 s), annealing temperature (30 s), and extension at 72°C (1 min). Method given by Livak and Schmittgen (2001) was referred to calculate fold change in gene expression from threshold values (*Ct*) obtained from qRT-PCR analysis (StepOne Software version 2.3, Applied Biosystems).

**Table 1:** **Primer sequences used for gene expression analysis using qRT-PCR**.

**Gene name**	**Primer sequence**	**Annealing temp. (°C)**	**Product size (bp)**
*Actin*	Forward primer 5′ CTTGCACCTAGCAGCATGAA 3′	52	154
	Reverse primer 5′ GGACAATGGATGGACCTGAC 3′		
*CS*	Forward primer 5′ TGGGACAGAGCTCTTGGACT 3′	51	140
	Reverse primer 5′ TCAGTGTGGAAGGAACACCA 3′		
*SUCLG1*	Forward primer 5′ ATTATGCCGGGTTACATCCA 3′	49	141
	Reverse primer 5′ AAAAGGATCCCCACCAATTC 3′		
*SDH*	Forward primer 5′ GTGGTCAGGCCTATCGTTGT 3′	54	154
	Reverse primer 5′ CCCTGGCAAGTACCATCACT 3′		
*FH*	Forward primer 5′ CTCTCCACCATCTCGTCTCC 3′	49	141
	Reverse primer 5′ CCCTGAACGAGGTCGAATAA 3′		
*MS*	Forward primer 5′ GGGCATGTGAGGTACGCTAT 3′	52	123
	Reverse primer 5′ AGAGGCACAAACCCATTCAC 3′		
*CHLASE*	Forward primer 5′ GAATATCCGGTGGTGATGCT 3′	49	161
	Reverse primer 5′ TCCGCCGTTGATTTTATCTC 3′		
*PSY*	Forward primer 5′ TGGGTTGGTAAGGGCTGTAG 3′	51	155
	Reverse primer 5′ CGCTCGAAGACACAACACTC 3′		
*CHS*	Forward primer 5′ CAAGGCGGAGAAGATGAGAG 3′	54	113
	Reverse primer 5′ CATCTTCCGCAGACTTCCTC 3′		
*PAL*	Forward primer 5′ AAACTCCGTCAACGACAACC 3′	54	142
	Reverse primer 5′ AGCGAACATGAGCTTCCCTA 3′		

*CS, citrate synthase; SUCLG1, succinyl Co-A ligase; SDH, succinate dehydrogenase; FH, fumarate hydratase; MS, malate synthase; CHLASE, chlorophyllase; PSY, phytoene synthase; CHS, chalcone synthase; and PAL, phenylalanine ammonialyase*.

### Statistical analysis

Statistical analysis of data was done using two-way ANOVA, Tukey's HSD, multiple linear regression (MLR) analysis (self-coded softwares in MS-excel 2010), and artificial neural networks (ANN) using Statistica-12 (Kumar et al., 2016; Sharma et al., 2016a,d).

## Results

### Effect of EBR seed soaking on growth parameters

As compared to control, seeding length and biomass (dry weight) was reduced by 420.78% and 220.68%, respectively under IMI toxicity (250 mg/L) over the control. However, seed soaking with EBR significantly enhanced the length of *B. juncea* seedlings by 179.21% and biomass by 137.93% grown under IMI stress (Table 2). Data analysis using two-way ANOVA and Tukey's HSD showed significant differences for seedling length (F_IMI_, F_EBR_, and F_IMI × EBR_ = *p* < 0.001) as well as dry weight (F_IMI_, F_EBR_, and F_IMI × EBR_ = *p* < 0.001) in *B. juncea* seedlings. Multiple linear regression (MLR) analysis of data also revealed the enhanced growth of seedlings raised from EBR soaked seeds grown under IMI stress. Negative β-regression coefficients for IMI showed decrease in seedling length and biomass as a consequence of IMI toxicity. Positive β-regression coefficients for EBR and interaction IMI × EBR showed recovery of the seedling growth, which was negatively affected by IMI toxicity (Table 2).

**Table 2:** **Effect of seed soaking with 24-epibrassinolide (EBR) on growth parameters in ***Brassica juncea*** seedlings grown under imidacloprid (IMI) toxicity**.

**Treatments**		**Seedling length (cm/seedling)**			**Dry weight (mg/seedling)**	
**IMI (mg/L)**	**24-EBR (nM)**					
0	0	14.53^ab^ ± 0.62			18.6^ab^ ± 2.17	
0	100	15.09^a^ ± 1.59			19.9^a^ ± 1.66	
150	0	10.87^c^ ± 1.14			16.1^abc^ ± 1.18	
150	100	13.71^ab^ ± 0.73			19.6^a^ ± 0.97	
200	0	6.76^d^ ± 0.80			9.2^d^ ± 0.91	
200	100	12.10^bc^ ± 0.60			15.4^bc^ ± 1.57	
250	0	2.79^e^ ± 0.22			5.8^d^ ± 0.92	
250	100	7.79^d^ ± 0.54			13.5^c^ ± 1.35	
**TWO-WAY ANOVA**						
F-ratios & HSD		F_IMI_ = 439^***^			F_IMI_ = 208^***^	
		F_EBR_ = 310^***^			F_EBR_ = 220^***^	
		F_IMI × EBR_ = 32.2^***^			F_IMI × EBR_ = 20.3^***^	
		HSD = 2.46^*^			HSD = 3.98^*^	
**Multiple linear regression equation**		**β regression coefficients**			**MLR**	**ANN**
		β_IMI_	β_EBR_	β_IMI × EBR_	***r***	***r*** **(validation)**
Seedling length (cm) = 15.38−0.03 X_1_ + 0.0042 X_2_ + 0.0001 X_1_X_2_		−0.9825	0.0522	0.4873	0.8984^**^	0.9549^***^
Dry weight (mg/seedling) = 19.77−0.033 X_1_ + 0.0088 X_2_ + 0.0002 X_1_X_2_		−0.9279	0.0929	0.5245	0.8801^**^	0.9666^***^

*Treatments with same superscripts indicates no significant difference at p < 0.05*,

*, **, and *** *indicate significant at p < 0.05, p < 0.01, and p < 0.001 respectively. X_1_, IMI; X_2_, EBR; r, correlation coefficient. Data are mean±standard deviation of 10 seedlings, two-way ANOVA, Tukey's HSD, multiple linear regression analysis and and artificial neural networks (ANN)*.

### Effect of EBR seed soaking on pigment system

**Figure 1:**
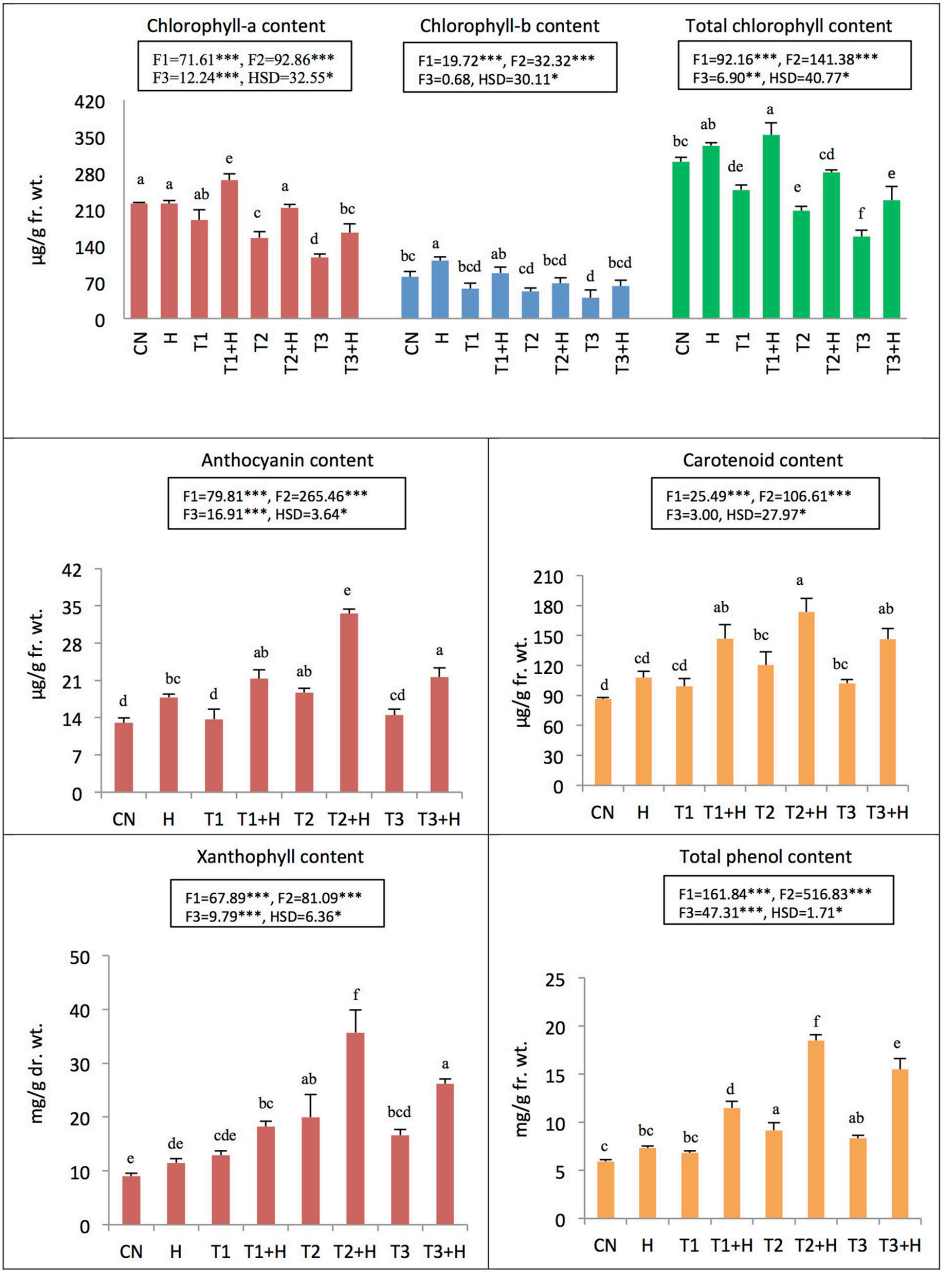
**Effect of seed soaking with 24-epibrassinolide (EBR) on pigment and phenol contents in ***Brassica juncea*** seedlings grown under imidacloprid (IMI) toxicity**. Data are mean ± standard deviation, two-way ANOVA, and Tukey's HSD (three biological replicates). Treatments with same letter indicates no significant difference at *p* < 0.05. F1, F2, and F3 are F-ratios for IMI, EBR, and IMI × EBR, respectively. CN, control; H, 100 nM EBR; T1, 150 mg IMI/L DW; T2, 200 mg IMI/L DW; and T3, 250 mg IMI/L DW. ^**^ and ^***^ indicate significant at *p* < 0.01 and *p* < 0.001, respectively.

Total chlorophyll content was decreased by 91.12% as a result of IMI toxicity (250 mg/L). However, seed soaking with 100 nM EBR resulted in the recovery of chlorophyll content by 44.13% under IMI stress (Figure 1). Furthermore, seedlings treated with EBR, grown under 200 mg IMI/L significantly enhanced the contents of carotenoids (101.10%), anthocyanins (157.55%), and xanthophylls (296.99%) in *B. juncea* seedlings, as compared to control (Figure 1). Two-way ANOVA and Tukey's HSD revealed that contents of pigments were significantly different in *B. juncea* seedlings under different treatments. MLR analysis revealed increase in the contents of pigments after EBR seed soaking. Concentration of IMI was negatively regressed upon chlorophyll content, indicating the degradation of chlorophyll under IMI stress. However, IMI showed positive regression with contents of carotenoids, anthocyanins, and xanthophylls, implying an increase in these pigments under IMI stress. EBR seed application was also regressed positively on the contents of all the pigments including chlorophyll-a, chlorophyll-b, total chlorophyll, carotenoids, anthocyanins, and xanthophylls. Moreover, it was observed that interactions between IMI and EBR were positive for all pigments studied except chlorophyll-b, where interaction (IMI × EBR) was noticed to be negative (**Table 4**).

### Effect of EBR on phenolic compounds

In the seedlings of *B. juncea*, nine polyphenols were detected (Table 3). It was observed that, as compared to control seedlings, the total polyphenols were increased by 101.22% in seedlings raised from 100 nM EBR treated seeds grown under IMI pesticide (200 mg/L) stress (Table 3). The total phenol content also enhanced by 213.56% with 100 nM RBR treated *B. juncea* under 200 mg/L IMI stress (Figure 1). Analysis of total polyphenol and total phenol contents using two-way ANOVA and Tukey's HSD also showed significant difference (F_IMI_, F_EBR_, and F_IMI × EBR_ = *p* < 0.001). MLR analysis also revealed that both IMI and EBR enhanced the content of total phenols. Concentrations of IMI as well as EBR were regressed positively on the content of total phenols. Additionally, positive interaction was observed between IMI and EBR for total phenol content (Table 4).

**Table 3:** **Effect of seed soaking with 24-epibrassinolide (EBR) on polyphenol contents in ***Brassica juncea*** seedlings grown under imidacloprid (IMI) toxicity**.

**TREATMENT**								
EBR (nM)	0	100	0	100	0	100	0	100
IMI (mg/L)	0	0	150	150	200	200	250	250
**POLYPHENOL CONTENT (μg/g fr. wt.)**								
Gallic acid	–	–	–	3.62 ± 0.17	–	–	–	–
Catechin	16.86 ± 3.39	62.15 ± 4.88	6.76 ± 1.09	25.31 ± 4.27	59.20 ± 7.59	206.17 ± 16.25	14.05 ± 2.43	16.93 ± 0.63
Chlorogenic acid	–	18.22 ± 2.32	8.74 ± 0.97	25.31 ± 5.37	16.10 ± 4.32	58.85 ± 7.53	4.81 ± 0.76	4.36 ± 0.65
Caffeic acid	50.05 ± 5.99	29.43 ± 4.14	49.30 ± 2.77	49.22 ± 8.71	30.35 ± 4.57	0.77 ± 0.12	104.71 ± 8.40	61.58 ± 5.32
Rutin	11.39 ± 2.12	–	24.28 ± 4.54	13.62 ± 3.43	–	13.11 ± 1.07	24.46 ± 4.78	31.76 ± 6.15
Ellagic acid	1.70 ± 0.18	–	5.98 ± 0.68	2.35 ± 0.11	–	–	2.50 ± 0.25	1.18 ± 0.03
tert-Butyl hydroquinone	0.15 ± 0.02	–	3.27 ± 0.39	0.09 ± 0.01	1.80 ± 0.04	3.05 ± 0.21	1.67 ± 0.22	0.31 ± 0.08
Quercetin	1.77 ± 0.14	0.02 ± 0.004	1.33 ± 0.11	1.16 ± 0.24	10.54 ± 1.23	–	2.86 ± 0.28	23.87 ± 3.31
Kaempferol	97.80 ± 10.22	89.20 ± 16.63	100.34 ± 11.53	98.18 ± 12.76	164.86 ± 31.81	80.03 ± 17.69	88.27 ± 18.58	216.33 ± 28.73
Total	179.7^c^ ± 21.19	199.0^c^ ± 19.62	199.9^c^ ±7.95	238.3^b^ ± 28.62	282.8^d^ ± 23.99	361.9^a^ ± 14.14	243.3^b^ ± 18.96	356.3^a^ ± 37.86
F-ratios and HSD for total polyphenol content, F_IMI_ = 456.66^***^, F_EBR_ = 34.556^***^, F_IMI × EBR_ = 34.555^***^, HSD = 23.33^*^								
**Multiple linear regression equation**				**β regression coefficients**			**MLR**	**ANN**
				**β_IMI_**	**β_EBR_**	**β_IMI × EBR_**	***r***	***r* (validation)**
Polyphenol content (μg/g fr. wt.) = 177.1 + 0.329 X_1_ + 0.0947 X_2_ + 0.0035 X_1_X_2_				0.4494	0.0691	0.5156	0.8619^***^	0.8738^***^

*Statistical analysis was done only for total polyphenol content. For individual polyphenol, statistical analysis was not done due to non-uniformity in their detection using HPLC. (−) means not detected. Treatments with same superscripts indicates no significant difference at p < 0.05. r, correlation coefficient*.

*, and *** *indicate significant at p < 0.05, and p < 0.001, X_1_, IMI; X_2_, EBR. Data are mean ± standard deviation (three biological replicates), two-way ANOVA, Tukey's HSD, multiple linear regression analysis (MLR) and artificial neural networks (ANN)*.

**Table 4:** **Multiple linear regression (MLR) and artificial neural network (ANN) analysis showing effect of seed soaking with 24-epibrassinolide (EBR) on pigments and total phenol contents, and gene expression in ***Brassica juncea*** seedlings grown under imidacloprid (IMI) toxicity**.

**Multiple linear regression equation**	**β regression coefficients**			**MLR**	**ANN**
	**β_IMI_**	**β_EBR_**	**β_IMI × EBR_**	***r***	***r* (validation)**
Chlorophyll-a content (μg/g fr. wt.) = 229.02 − 0.258 X_1_ + 0.0707 X_2_ + 0.0017 X_1_X_2_	−0.7791	0.0792	0.5611	0.7724^***^	0.9960^***^
Chlorophyll-b content (μg/g fr. wt.) = 81.29 − 0.106 X_1_ + 0.3187 X_2_ − 3 × 10^−4^ X_1_X_2_	−0.6259	0.7006	−0.2062	0.9068^***^	0.9837^***^
Total chlorophyll content (μg/g fr. wt.) = 310.24 − 0.364 X_1_ + 0.3892 X_2_ + 0.0014 X_1_X_2_	−0.7807	0.3102	0.3241	0.8562^***^	0.9937^***^
Carotenoid content (μg/g fr. wt.) = 86.90 + 0.0669 X_1_ + 0.2345 X_2_ + 0.0008 X_1_X_2_	0.3092	0.4026	0.4101	0.8910^***^	0.9158^***^
Anthocyanin content (μg/g fr. wt.) = 13.05 + 0.0086 X_1_ + 0.0512 X_2_ + 0.0002 X_1_X_2_	0.1841	0.4064	0.3601	0.7829^***^	0.9728^***^
Xanthophyll content (mg/g dr. wt.) = 8.80 + 0.0258 X_1_ + 0.0224 X_2_ + 0.0003 X_1_X_2_	0.4168	0.1339	0.4737	0.8327^***^	0.9134^***^
Total phenol content (μg/g fr. wt.) = 5.79 + 0.0078 X_1_ + 0.013 X_2_ + 0.0002 X_1_X_2_	0.2485	0.1536	0.6729	0.9213^***^	0.9066^***^
*CS* (gene expression fold change) = 1 + 0.0068 X_1_ + 0.0277 X_2_ - 1 × 10^−4^ X_1_X_2_	0.6281	1.2822	−1.007	0.9117^***^	0.8090^***^
*SUCLG1* (gene expression fold change) = 1 + 0.0029 X_1_ + 0.0.0372 X_2_ − 6 × 10^−5^ X_1_X_2_	0.1781	1.1436	−0.2962	0.9875^***^	0.9870^***^
*SDH* (gene expression fold change) = 1 + 0.0051 X_1_ + 0.0199 X_2_ − 7 × 10^−5^ X_1_X_2_	0.5806	1.1351	−0.7171	0.8480^**^	0.5441^***^
*FH* (gene expression fold change) = 1 + 0.0029 X_1_ + 0.0318 X_2_ − 5 × 10^−5^ X_1_X_2_	0.2068	1.1372	−0.3193	0.9707^***^	0.9475^***^
*MS* (gene expression fold change) = 1 + 0.0046 X_1_ + 0.0289 X_2_ − 4 × 10^−5^ X_1_X_2_	0.3322	1.0422	−0.2403	0.9342^***^	0.9489^***^
*CHLASE* (gene expression fold change) = 1 + 0.0083 X_1_ + 0.0026 X_2_ − 9 × 10^−5^ X_1_X_2_	1.1463	0.1788	−1.1018	0.9349^***^	0.8785^***^
*PSY* (gene expression fold change) = 1 + 0.0136 X_1_ + 0.0028 X_2_ + 6 × 10^−5^ X_1_X_2_	0.7504	0.0771	0.2964	0.9697^***^	0.9362^***^
*CHS* (gene expression fold change) = 1 + 0.0166 X_1_ + 0.0038 X_2_ − 8 × 10^−6^ X_1_X_2_	0.9839	0.1116	−0.0415	0.9642^***^	0.9063^***^
*PAL* (gene expression fold change) = 1 + 0.0049 X_1_ + 0.0112 X_2_ + 0.0002 X_1_X_2_	0.2065	0.2383	0.6588	0.9338^***^	0.9207^***^

*CS, citrate synthase; SUCLG1, succinyl Co-A ligase; SDH, succinate dehydrogenase; FH, fumarate hydratase; MS, malate synthase; CHLASE, chlorophyllase; PSY, phytoene synthase; CHS, chalcone synthase; and PAL, phenylalanine ammonialyase*.

** and *** *indicate significant at p < 0.01 and p < 0.001. r, correlation coefficient; X_1_, IMI; X_2_, EBR*.

### Effect of EBR on organic acids

The contents of organic acids *viz*. fumarate, succinate, malate, and citrate were observed to enhance by 3.31, 27.05, 328.06, and 63.86% in the seedlings raised from EBR treated/untreated seeds grown under IMI pesticide stress, when compared to control seedlings (Table 5). Significant differences in the contents organic acids including fumarate (F_IMI_ = *p* < 0.001, F_EBR_ = *p* < 0.001, and F_IMI × EBR_ = *p* < 0.05), succinate (F_IMI_ = *p* < 0.001 and F_EBR_ = *p* < 0.001), malate (F_IMI_ = *p* < 0.001, F_EBR_ = *p* < 0.001, and F_IMI × EBR_ = *p* < 0.01), and citrate (F_IMI_ = *p* < 0.001, F_EBR_ = *p* < 0.001) were observed after analyzing the data using two-way ANOVA and Tukey's HSD (Table 5). Positive β-regression coefficients obtained from MLR analysis of the contents of organic acids also revealed that both IMI as well as EBR resulted in increase in the contents of organic acids in *B. juncea* seedlings. However, β_IMI × EBR_ revealed that there were negative interactions between IMI and EBR for the contents of fumarate, succinate, and citrate, whereas positive interaction between IMI and EBR was noticed for malate content (Table 5).

**Table 5:** **Effect of seed soaking with 24-epibrassinolide (EBR) on contents of organic acids in ***Brassica juncea*** seedlings grown under imidacloprid (IMI) toxicity**.

**Treatments**		**Fumarate (mg/g DW)**	**Succinate (mg/g DW)**	**Malate (mg/g DW)**	**Citrate (mg/g DW)**		
**IMI (mg/L)**	**24-EBR (nM)**						
0	0	0.3709^d^±0.0006	0.85^d^±0.01	1.39^c^±0.06	3.21^c^±0.45		
0	100	0.3746^bcd^±0.0003	0.96^bc^±0.04	1.47^c^±0.08	3.86^bc^±0.12		
150	0	0.3727^cd^±0.0032	0.94^bcd^±0.05	2.81^bc^±0.31	3.81^bc^±0.31		
150	100	0.3765^bc^±0.0030	1.00^ab^±0.04	5.94^a^±1.77	4.32^b^±0.31		
200	0	0.3757^bcd^±0.0012	0.97^b^±0.02	2.82^bc^±0.05	4.37^b^±0.21		
200	100	0.3832^a^±0.0019	1.08^a^±0.04	5.91^a^±1.03	5.26^a^±0.51		
250	0	0.3782^b^±0.0008	0.86^d^±0.03	3.29^bc^±0.07	3.40^c^±0.08		
250	100	0.3784^ab^±0.0002	0.88^cd^±0.02	4.59^ab^±0.46	3.80^bc^±0.29		
**TWO-WAY ANOVA**							
F-ratios & HSD		F_IMI_ = 18.3^***^	F_IMI_ = 24.7^***^	F_IMI_ = 21.1^***^	F_IMI_ = 20.8^***^		
		F_EBR_ = 27.3^***^	F_EBR_ = 27.8^***^	F_EBR_ = 38.3^***^	F_EBR_ = 22.5^***^		
		F_IMI × EBR_ = 4.09^*^	F_IMI × EBR_ = 2.16	F_IMI × EBR_ = 5.84^**^	F_IMI × EBR_ = 0.67		
		HSD = 0.005^*^	HSD = 0.089^*^	HSD = 2.13^*^	HSD = 0.85^*^		
**Multiple linear regression equation**			**β regression coefficients**			**MLR**	**ANN**
			**β_IMI_**	**β_EBR_**	**β_IMI × EBR_**	**r**	**r (validation)**
Fumarate (mg/g dr. wt.) = 0.37 + 3 × 10^−5^ X_1_ + 5 × 10^−5^ X_2_ − 5 × 10^−8^ X_1_X_2_			0.6722	0.5846	−0.1166	0.7947^***^	0.4726^***^
Succinate (mg/g dr. wt.) = 0.87 + 0.0002 X_1_ + 0.0011 X2 − 2 × 10^−6^ X_1_X_2_			0.2256	0.6938	−0.2975	0.4983^*^	0.8787^***^
Malate (mg/g dr. wt.) = 1.46 + 0.0075 X_1_ + 0.0073 X_2_ + 8 × 10^−5^ X_1_X_2_			0.3906	0.2050	0.4349	0.8234^***^	0.9633^***^
Citrate (mg/g dr. wt.) = 3.36 + 0.0022 X_1_ + 0.0067 X_2_ − 4 × 10^−6^ X_1_X_2_			0.3155	0.5119	−0.0589	0.5499^**^	0.9236^***^

*Treatments with same superscripts indicates no significant difference at p < 0.05*.

*, **, and *** *indicate significant at p < 0.05, p < 0.01, and p < 0.001 respectively. r, correlation coefficient; X_1_, IMI; X_2_, EBR. Data are mean ± standard deviation (three biological replicates), two-way ANOVA, Tukey's HSD, multiple linear regression analysis and and artificial neural networks (ANN)*.

### Gene expression

**Figure 2:**
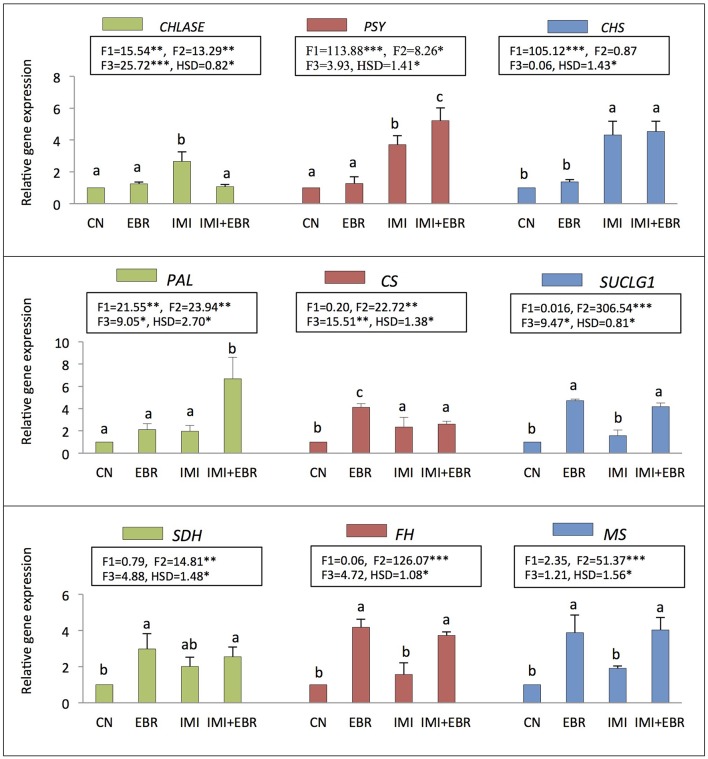
**Effect of seed soaking with 24-epibrassinolide (EBR) on gene expression of ***Brassica juncea*** seedlings grown under imidacloprid (IMI) toxicity**. Data are mean ± standard deviation, two-way ANOVA and Tukey's HSD (three biological replicates). Treatments with same letter indicates no significant difference at *p* < 0.05. F1, F2, and F3 are F-ratios for IMI, EBR, and IMI × EBR, respectively. CN, control; EBR, 100 nM EBR; IMI, 200 mg IMI/L DW; *CS*, citrate synthase; *SUCLG1*, succinyl Co-A ligase; *SDH*, succinate dehydrogenase; *FH*, fumarate hydratase; *MS*, malate synthase; *CHLASE*, chlorophyllase; *PSY*, phytoene synthase; *CHS*, chalcone synthase; and *PAL*, phenylalanine ammonialyase. ^*^, ^**^, and ^***^ indicate significant at *p* < 0.05, *p* < 0.01, and *p* < 0.001, respectively.

In the present study, as compared to control seedlings, the expression of gene *CHLASE* (encoding chlorophyllase) was observed to increase by 2.66-fold under IMI toxicity, but seed soaking with EBR significantly reduced the expression of *CHLASE* to 1.07-fold in the seedlings under IMI toxicity (Figure 2). Data analysis using two-way ANOVA and Tukey's HSD showed significant difference for *CHLASE* expression in *B. juncea* seedlings (F_IMI_ *p* < 0.01, F_EBR_ *p* < 0.01, F_IMI × EBR_ *p* < 0.001). MLR analysis of the fold change in *CHLASE* expression also revealed the increased expression of gene with IMI toxicity and EBR application (positive β_IMI_-value), whereas interaction between IMI and EBR was observed to be negative (Table 4).

Further, in comparison to control seedlings, the expression of *PSY* (encoding phytoene synthase) and *CHS* (encoding chalcone synthase) was significantly enhanced by 5.22 and 4.54-folds respectively in the seedlings raised from EBR treated as well as untreated seeds grown under IMI stress (Figure 2). Significant differences in expression *PSY* (F_IMI_ *p* < 0.001, F_EBR_ *p* < 0.05) and *CHS* (F_IMI × EBR_ *p* < 0.001) were observed after analyzing the data using two-way ANOVA and Tukey's HSD. MLR analysis of fold change in gene expression also revealed the role of EBR in modulation of gene expression of *PSY* and *CHS*. Concentrations of IMI as well as EBR were regressed positively on the fold change in gene expression of *PSY* and *CHS*, thus revealing enhanced expressions of these genes under both the treatments. Moreover, interaction between IMI and EBR was positive for *PSY* expression, whereas negative interaction was observed for the expression of *CHS* (Table 4).

In the present study, the expression of *PAL* was also observed to enhance significantly by 6.68-fold in the seedlings raised from EBR treated seeds and grown under IMI stress (Figure 2). After analyzing the data using two-way ANOVA and Tukey's HSD, significant difference in the expression of *PAL* was observed (F_IMI_ *p* < 0.01, F_EBR_ *p* < 0.01, F_IMI × EBR_ *p* < 0.05). MLR analysis of the fold change in gene expression also confirmed the role of EBR in increasing the *PAL* gene expression under IMI pesticide stress. Positive β-regression coefficients were observed for IMI, EBR, and IMI × EBR (Table 4).

The expression of genes encoding the key enzymes involved in organic acid metabolism was also studied to understand the role of EBR in organic acid metabolism under IMI pesticide stress. It was observed that as compared to control seedlings, the expression of *CS* (encoding citrate synthase, 2.35-fold), *SUCLG1* (encoding succinyl-Co-A ligase, 1.57-fold), *SDH* (encoding succinate dehydrogenase, 2.01-fold), *FH* (encoding fumarate hydratase, 1.57-fold), and *MS* (encoding malate synthase, 1.91-fold) were increased in *B. juncea* seedlings raised from untreated seeds and grown under IMI pesticide toxicity (Figure 2). However, seed soaking with 100 nM EBR and germinating them under IMI toxicity resulted in further enhancement in expression of *CS* (2.61-fold), *SUCLGD1* (4.18-fold), *SDH* (2.55-fold), *FH* (3.73-fold), and *MS* (4.03-fold). Data analysis using two-way ANOVA and Tukey's HSD showed significant differences in the expression of *CS* (F_EBR_ *p* < 0.01, F_IMI × EBR_ *p* < 0.01), *SUCLG1* (F_EBR_ *p* < 0.001, F_IMI × EBR_ *p* < 0.05), *SDH* (F_EBR_ *p* < 0.01), *FH* (F_EBR_ *p* < 0.001), and *MS* (F_EBR_ *p* < 0.001). MLR analysis showed that gene expression in seedlings under IMI stress as well as after the EBR seed treatment was increased as indicated by positive β-regression coefficients. Whereas, negative interactions were noticed between IMI and EBR treatments for the expression of all genes studied related to organic acid metabolism (Table 4).

### Analysis of data using artificial neural networks (ANN)

From ANN analysis of data, it has been observed that correlation between experimental values and simulated values for all the parameters studied against, using EBR and IMI as inputs are highly correlated (Tables 2–4). This revealed that ANN can simulate the experimental data with high level of significance. Earlier studies have also reported high correlations for enzymatic antioxidants, and pesticide residues against applied IMI and EBR (Sharma et al., 2016c).

## Discussion

In the present study, seed soaking with EBR resulted in recovery of the growth of *B. juncea* seedlings raised under IMI toxicity. The enhancement in seedling growth after the application of EBR might be due to the ability of BRs to modulate cellulose biosynthesis, along with cell division and cell elongation (González-García et al., 2011; Hacham et al., 2011; Xie et al., 2011). Increase in growth parameters after the exogenous application of BRs has also been reported by Sharma et al. (2015) in rice seedlings.

Decrease in chlorophyll contents might due to the degradation of chloroplast, oxidation of chlorophylls as a result of oxidative stress and enhanced activity of chlorophyllase enzyme (Kato and Shimizu, 1985; Parida et al., 2002; Harpaz-Saad et al., 2007). Moreover, it has also been reported that BRs up-regulate the transcripts and process of translation during chlorophyll biosynthesis and reduction of chlorophyll degradation (Kalinich et al., 1985; Honnerova et al., 2010). In the present study, it has been observed that EBR reduced the expression of *CHLASE* under IMI stress, suggesting the possible reason for recovery of chlorophyll contents in *B. juncea* seedlings grown under IMI stress.

Increase in carotenoid, xanthophyll, and anthocyanin contents has been observed with the application of EBR under IMI stress. Since phytoene synthase (PSY) is a key enzyme in the biosynthetic pathway of carotenoids and xanthophylls, the change in the expression of *PSY* could be one of the reasons for alterations in the contents of carotenoids and xanthophylls. In the present study, the expression of *PSY* as well as the contents of carotenoids and xanthophylls were observed to enhance in seedlings raised from EBR treated seeds and grown under IMI stress. Chalcone synthase (*CHS*) plays an important role in the biosynthetic pathway of anthocyanins, and in the present experiment, the expression of *CHS* was observed to increase in *B. juncea* seedlings raised from EBR treated seeds grown in IMI solutions. The enhanced contents of anthocyanins in *B. juncea* seedlings might be due to the modulation of *CHS* by EBR. Additionally, Luan et al. (2013) also reported that BRs up-regulate the genes which are responsible for the biosynthesis of anthocyanins. Moreover, BRs have also been reported to induce anthocyanin biosynthesis by BRs-cytokinin mediated regulation of late anthocyanin biosynthetic genes (Yuan et al., 2015).

The contents of phenolic compounds were enhanced in the present study with the application of IMI as well as EBR. The enhanced levels of polyphenols and total phenolic contents might be due to the stress-induced activation of phenylpropanoid pathway (Korkina, 2007). Further, in the present study, application of EBR also increased the activity of phenylalanine ammonialyase (PAL), a key enzyme of phenylpropanoid pathway (Ahammed et al., 2013; Xi et al., 2013). The results of present study are in agreement with the studies carried out by Siddiqui and Ahmed (2006). They reported the enhanced contents of total phenols in soybean plants under pesticide stress. BRs have also been reported to increase the total phenol contents in *Vitis vivifera L*. (Champa et al., 2015) and in *Cichorium endivia L*. (Serna et al., 2013).

In the present experiment, contents of organic acids studied were increased after the application of IMI and EBR. It is well known that citrate synthase catalyses the synthesis of citrate, succinyl-Co-A ligase catalyses the synthesis of succinate, succinate dehydrogenase catalyses the synthesis of fumarate, and fumarate hydratase and malate synthase catalyses the synthesis of malate (Lehninger et al., 2008). The EBR mediated regulation of the genes (*CS, SUCLG1, SDH, FH*, and *MS*) encoding enzymes involved in organic acid metabolism might be a possible reason of increased contents of all the organic acids under IMI toxicity. Moreover, the biosynthesis of organic acids in plants has been reported to get enhanced under abiotic stress conditions (Timpa et al., 1986; Li et al., 2000; Ma, 2000). The present study also reported increase in organic acid contents under pesticide stress, and seed soaking with EBR further enhanced their levels, proposing the role of these organic acids in ameliorating pesticide toxicity.

In the present study, BR-modulated mitigation of IMI toxicity might be due to the BR-signaling which regulated the expression of genes studied in the present work. It is well known that BR signaling starts with BRI1 (BRASSINOSTEROID SENSTIVE 1) and its co-receptor BAK1 (BRI1-associated receptor kinase 1; Hao et al., 2013). BKI1 (BRI1 KINASE INHIBTOR 1) has been reported to undergo tyrosine phosphorylation as a result of BR signaling (Jaillais et al., 2011). Moreover, CaM (calmodulin) binding to BRI1 and DWF4 (DWARF4) has also been reported in Ca^2+^ dependent manner which is supposed to be an important step in BR signaling (Du and Poovaiah, 2005; Oh et al., 2012). After receiving BR signal, process of phosphorylation and dephosphorylation leads to stimulation of transcription factors (TFs) which control BR-mediated gene expression, involving BES1 (BRI1-EMS-Supressor 1) and BZR (Brasssinozole resistant 1). These TFs are regulated by BIN2 (BRASSINOSTEROID INSENSTIVE 2), BSK1 (BR-SIGANALLING KINASE 1), BSU1 (BRIL SUPPRESSOR 1), and PP2A (PROTEIN PHOSPHATE 2A; Wang et al., 2002; He et al., 2005; Yin et al., 2005; Ye et al., 2011). It further results in modulation of various biological processes leading to regulation of vegetative as well as reproductive development of plants (Clouse, 2011). Studies have also reported that phosphorylation and dephosphorylation of casein kinase2 and MAPK (mitogen activated protein kinase) substrates were observed after the application of BR (Lin et al., 2015). Moreover, earlier studies have also demonstrated that BRs interact with other plant hormones to regulate the growth and development of plants (Choudhary et al., 2012). Binding of BZR1 protein to promoter regions of *IAA19* and *ARF7* regulates plant growth and development as a result of BR-auxin crosstalk (Zhou et al., 2013). Pollen tube cell expansion has been reported to be regulated by BR-ethylene crosstalk by modulation of *FERONIA*, which encodes a receptor like kinase involved in pollen tube development (Huck et al., 2003; Escobar-Restrepo et al., 2007). In rice plants, enhancement in content of cytokinins was observed under drought stress after the up-regulation of BR related genes like *DWF5, BAK1, BSK1*, and *SERK1* suggesting BRs-CKs signaling (Peleg et al., 2011). BR homeostasis is also necessary in plants for normal biological functions. It is regulated by the feedback expression of various genes involved in the biosynthesis of BRs as well as sterols (Tanaka et al., 2005). These researchers demonstrated that BZR application has been resulted in enhancing the expression of BR-biosynthetic genes like *DWF4, CONSTITUTIVE PHOTOMORPHOGENESIS AND DWARFISM (CPD), DEETIOLATED2 (DET2), BR-6-oxidase (BR6ox1)*, and *ROTUNDFOLIA3 (ROT3)* in BR-depleted *Arabidopsis* plants. However, the exogenous application of BL resulted in the down-regulation of *DWF4, CPD, BR6ox1*, and *ROT3*. Esterification process by putative CoA-dependent acyltransferases encoded by *DRL1* (*DWARF AND ROUND LEAF-1*) which plays an important role in BR homeostasis (Zhu et al., 2013).

## Conclusions and future prospects

From the present study it may be concluded that seed soaking with 24-epibrassinolide recovers the impaired growth of *B. juncea* seedlings under imidacloprid stress by modulating the expression of genes encoding key enzymes including chlorophyllase, citrate synthase, succinyl Co-A ligase, succinate dehydrogenase, fumarate hydratase, malate synthase, phytoene synthase, chalcone synthase, and phenylalanine ammonialyase. In future studies, the expression analysis of BR specific biosynthetic genes including *DWF4, CPD, DET2, BR6ox1*, and *ROT3* in pesticide stressed plants would help in understanding the mechanisms of BR mediated pesticide detoxification. In addition to this *BRI1* silencing and studying the expression of CaM encoding genes would help in understanding the initial steps of BR signaling. Moreover, total transcriptome sequencing and microarray analysis and total phosphoproteome profiling of plants germinated from BR soaked seeds and grown in presence of pesticides could help in exploring the detailed mechanisms of BR-mitigated pesticide toxicity.

## Author contributions

AS, ST, and MK performed the experimental work and also helped in writing of this manuscript. VK, AT, and PAl analyzed the data. RB, AT, AK, and PAh designed the experimental work, evaluated the results, and wrote and revised the manuscript.

### Conflict of interest statement

The authors declare that the research was conducted in the absence of any commercial or financial relationships that could be construed as a potential conflict of interest.

## References

[B1] Ahammed G. J. Zhou Y. H. Xia X. J. Mao W. H. Shi K. Yu J. Q. (2013). Brassinosteroid regulates secondary metabolism in tomato towards enhanced tolerance to phenanthrene. Biol. Plant. 57, 154–158. 10.1007/s10535-012-0128-9

[B2] Arnon D. I. (1949). Copper enzymes in isolated chloroplasts. Polyphenoloxidase in Beta vulgaris. Plant Physiol. 24, 1–15. 10.1104/pp.24.1.1 16654194 PMC437905

[B3] Bonmatin J. M. Marchand P. A. Charvet R. Moineau I. Bengsch E. R. Colin M. E. (2005). Quantification of imidacloprid uptake in maize crops. J. Agric. Food Chem. 53, 5336–5341. 10.1021/jf0479362 15969515

[B4] Champa W. H. Gill M. I. S. Mahajan B. V. C. Aror N. K. Bedi S. (2015). Brassinosteroids improve quality of table grapes (*Vitis vinifera* L.) cv. flame seedless. Trop. Agric. Res. 26, 368–379. 10.4038/tar.v26i2.8099 PMC4444924 26028743

[B5] Chen M. C. Wang M. K. Chiu C. Y. Huang P. M. King H. B. (2001). Determination of low molecular weight dicarboxylic acids and organic functional groups in rhizosphere and bulk soils of Tsuga and Yushania in a temperate rain forest. Plant Soil 231, 37–44. 10.1023/A:1010347421351

[B6] Chen X. Li W. Lu Q. Wen X. Li H. Kuang T.. (2011). The xanthophyll cycle and antioxidative defense system are enhanced in the wheat hybrid subjected to high light stress J. Plant Physiol. 168, 1828–1836. 10.1016/j.jplph.2011.05.019 21737175

[B7] Choudhary S. P. Yu J. Q. Yamaguchi-Shinozaki K. Shinozaki K. Tran L. S. P. (2012). Benefits of brassinosteroid crosstalk. Trends Plant Sci. 17, 594–605. 10.1016/j.tplants.2012.05.012 22738940

[B8] Clouse S. D. (2011). Brassinosteroid signal transduction: from receptor kinase activation to transcriptional networks regulating plant development. Plant Cell 23, 1219–1230. 10.1105/tpc.111.084475 21505068 PMC3101532

[B9] Ding H. Wen D. Fu Z. Qian H. (2014). The secretion of organic acids is also regulated by factors other than aluminum. Environ. Monit. Assess. 186, 1123–1131. 10.1007/s10661-013-3443-5 24097010

[B10] Du L. Poovaiah B. W. (2005). Ca^2+^/calmodulin is critical for brassinosteroids biosynthesis and plant growth. Nature 437, 741–745. 10.1038/nature03973 16193053

[B11] El-Naggar J. B. Zidan N. E. H. A. (2013). Field evaluation of imidacloprid and thiamethoxam against sucking insects and their side effects on soil fauna. J. Plant Prot. Res. 53, 375–387. 10.2478/jppr-2013-0056

[B12] Escobar-Restrepo J. M. Huck N. Kessler S. Gagliardini V. Gheyselinck J. Yang W. C. (2007). The FERONIA receptor-like kinase mediates male- female interactions during pollen tube reception. Science 317, 656–660. 10.1126/science.1143562 17673660

[B13] González-García M. P. Vilarrasa-Blasi J. Zhiponova M. Divol F. Mora-García S. Russinova E.. (2011). Brassinosteroids control meristem size by promoting cell cycle progression in Arabidopsis roots. Development 138, 849–859. 10.1242/dev.057331 21270057

[B14] Hacham Y. Holland N. Butterfield C. Ubeda-Tomas S. Bennett M. J. Chory J.. (2011). Brassinosteroid perception in the epidermis controls root meristem size. Development 138, 839–848. 10.1242/dev.061804 21270053 PMC3035089

[B15] Hao J. Yin Y. Shui-zhang F. (2013). Brassinosteroid signaling network: implications on yield and stress tolerance. Plant Cell Rep. 32, 1017–1030. 10.1007/s00299-013-1438-x 23568410

[B16] Harpaz-Saad S. Azoulay T. Arazi T. Yaakov E. B. Mett A. Shiboleth Y. M.. (2007). Chlorophyllase is a rate-limiting enzyme in chlorophyll catabolism and is post translationally regulated. Plant Cell 19, 1007–1022. 10.1105/tpc.107.050633 17369368 PMC1867358

[B17] Hayat S. Hasan S. A. Yusuf M. Hayat Q. Ahmad A. (2010). Effect of 28-homobrassinolide on photosynthesis, fluorescence and antioxidant system in the presence or absence of salinity and temperature in *Vigna radiata*. Environ. Exp. Bot. 69, 105–112. 10.1016/j.envexpbot.2010.03.004

[B18] He J. X. Gendron J. M. Sun Y. Gampala S. S. Gendron N. Sun C. Q.. (2005). BZR1 is a transcriptional repressor with dual roles in brassinosteroid homeostasis and growth responses. Science 307, 1634–1638. 10.1126/science.1107580 15681342 PMC2925132

[B19] Honnerova J. Rothova O. Hola D. Kocova M. Kohout L. Kvasnica M. (2010). The exogenous application of brassinosteroids to *Zea mays* (L) stressed by long term chilling does not affect the activities of photosystem 1 or 2. J. Plant Growth Regul. 29, 500–505. 10.1007/s00344-010-9153-027784017

[B20] Huck N. Moore J. M. Federer M. Grossniklaus U. (2003). The *Arabidopsis* mutant feronia disrupts the female gametophytic control of pollen tube reception. Development 130, 2149–2159. 10.1242/dev.00458 12668629

[B21] Jaillais Y. Hothorn M. Belkhadir Y. Dabi T. Nimchuk Z. L. Meyerowitz E. M.. (2011). Tyrosine phosphorylation controls brassinosteroids receptor activation by triggering membrane release of its kinase inhibitor. Genes Dev. 25, 232–237. 10.1101/gad.2001911 21289069 PMC3034898

[B22] Kalinich J. F. Mandava N. B. Todhunter J. A. (1985). Relationship of nucleic acid metabolism to brassinolide-induced responses in beans. J. Plant Physiol. 120, 207–214. 10.1016/S0176-1617(85)80107-3

[B23] Kapoor D. Kaur S. Bhardwaj R. (2014). Physiological and biochemical changes in *Brassica juncea* plants under Cd-induced stress. Biomed. Res. Int. 2014:726070. 10.1155/2014/726070 25133178 PMC4123575

[B24] Kato M. Shimizu S. (1985). Chlorophyll metabolism in higher plants VI. Involvement of peroxidase in chlorophyll degradation. Plant Cell Physiol. 26, 1291–1301.

[B25] Kilic S. Duran R. E. Coskun Y. (2015). Morphological and physiological responses of maize (*Zea mays* L.) seeds grown under increasing concentrations of chlorantraniliprole insecticide. Pol. J. Environ. Stud. 24, 1069–1075. 10.15244/pjoes/31339

[B26] Ko A. Y. Rahman M. M. Abd El-Aty A. M. Jang J. Park J. H. Cho S. K.. (2014). Development of a simple extraction and oxidation procedure for the residue analysis of imidacloprid and its metabolites in lettuce using gas chromatography. Food Chem. 148, 402–409. 10.1016/j.foodchem.2013.10.055 24262575

[B27] Korkina L. G. (2007). Phenylpropanoids as naturally occurring antioxidants: from plant defense to human health. Cell Mol. Biol. 53, 15–25. 10.1170/T772 17519109

[B28] Kumar V. Sharma A. Chawla A. Bhardwaj R. Thukral A. K. (2016). Water quality assessment of river Beas, India, using multivariate and remote sensing techniques. Environ. Monit. Assess. 188, 137. 10.1007/s10661-016-5141-6 26842241

[B29] Lawrence J. F. (1990). Determination of total xanthophyll and marigold oleoresin. J. Ass. Off. Anal. Chem. 2, 970–975.

[B30] Lehninger A. L. Nelson D. L. Cox M. M. (2008). Lehninger Principles of Biochemistry, 5th Edn. New York, NY: W.H. Freeman.

[B31] Li X. F. Ma J. F. Matsumoto H. (2000). Pattern of aluminum-induced secretion of organic acids differs between rye and wheat. Plant Physiol. 123, 1537–1544. 10.1104/pp.123.4.1537 10938369 PMC59110

[B32] Lin L. L. Hsu C. L. Hu C. W. Ko S. Y. Hsieh H. L. Huang H. C.. (2015). Integrating phosphoproteomics and bioinformatics to study brassinosteroid-regulated phosphorylation dynamics in *Arabidopsis*. BMC Genomics 16:533. 10.1186/s12864-015-1753-4 26187819 PMC4506601

[B33] Livak K. J. Schmittgen T. D. (2001). Analysis of relative gene expression data using real-time quantitative PCR and the 2^−ΔΔCT^ method. Methods 25, 402–408. 10.1006/meth.2001.1262 11846609

[B34] Luan L. Y. Zhang Z. W. Xi Z. M. Huo S. S. Ma L. N. (2013). Brassinosteroids regulate anthocyanin biosynthesis in the ripening of grape berries. S. Afr. J. Enol. Vitic. 34, 196–203.

[B35] Ma J. F. (2000). Role of organic acids in detoxification of aluminum in higher plants. Plant Cell Physiol. 41, 383–390. 10.1093/pcp/41.4.383 10845450

[B36] Mancinelli A. L. (1984). Photoregulation of anthocyanin synthesis VIII. Effect of light pretreatments. Plant Physiol. 75, 447–453. 10.1104/pp.75.2.447 16663641 PMC1066927

[B37] Nakabayashi R. Yonekura-Sakakibara K. Urano K. Suzuki M. Yamada Y. Nishizawa T.. (2014). Enhancement of oxidative and drought tolerance in Arabidopsis by overaccumulation of antioxidant flavonoids. Plant J. 77, 367–379. 10.1111/tpj.12388 24274116 PMC4282528

[B38] Oh M. H. Kim H. S. Wu X. Clouse S. D. Zielinski R. E. Huber S. C. (2012). Calcium/calmodulin inhibition of the *Arabidopsis* BRASSINOSTEROID-INSENSITIVE 1 receptor kinase provides a possible link between calcium and brassinosteroid signalling. Biochem. J. 443, 515–523. 10.1042/BJ20111871 22309147 PMC3316158

[B39] Parida A. Dam A. B. Dam P. (2002). NaCl stress causes changes in photosynthetic pigments proteins and other metabolic components in the leaves of a true mangrove *Bruguiera parviflora* in hydroponic cultures. J. Plant Biol. 45, 28–36. 10.1007/BF03030429

[B40] Peleg Z. Reguera M. Tumimbang E. Walia H. Blumwald E. (2011). Cytokinin-mediated source/sink modifications improve drought tolerance and increase grain yield in rice under water-stress. Plant Biotechnol. J. 9, 747–758. 10.1111/j.1467-7652.2010.00584.x 21284800

[B41] Serna M. Hernández F. Coll F. Coll Y. Amorós A. (2013). Effects of brassinosteroid analogues on total phenols, antioxidant activity, sugars, organic acids and yield of field grown endive (*Cichorium endivia L*.). J. Sci. Food Agric. 93, 1765–1771. 10.1002/jsfa.5968 23184906

[B42] Sharma A. Bhardwaj R. Kumar V. Thukral A. K. (2016c). GC-MS studies reveal stimulated pesticide detoxification by brassinolide application in *Brassica juncea* L. plants. Environ. Sci. Pollut. Res. 23, 14518–14525. 10.1007/s11356-016-6650-0 27068909

[B43] Sharma A. Kumar V. Bhardwaj R. Thukral A. K. (2016d). Seed pre-soaking with 24-epibrassinolide reduces the imidacloprid pesticide residues in green pods of *Brassica juncea* L. Toxicol. Environ. Chem. 1–9. 10.1080/02772248.2016.1146955 27454204

[B44] Sharma A. Kumar V. Singh R. Thukral A. K. Bhardwaj R. (2016b). Effect of seed pre-soaking with 24-epibrassinolide on growth and photosynthetic parameters of *Brassica juncea* L. in imidacloprid soil. Ecotoxicol. Env. Saf. 133, 195–201. 10.1016/j.ecoenv.2016.07.008 27454204

[B45] Sharma A. Kumar V. Thukral A. K. Bhardwaj R. (2016a). Epibrassinolide-imidacloprid interaction enhances non-enzymatic antioxidants in *Brassica juncea* L. Ind. J. Plant Physiol. 21, 70–75. 10.1007/s40502-016-0203-x

[B46] Sharma I. Bhardwaj R. Pati P. K. (2012). Mitigation of adverse effects of chlorpyrifos by 24-epibrassinolide and analysis of stress markers in a rice variety Pusa Basmati-1. Ecotoxicol. Environ. Saf. 85, 72–81. 10.1016/j.ecoenv.2012.07.003 22939030

[B47] Sharma I. Bhardwaj R. Pati P. K. (2013). Stress modulation response of 24-epibrassinolide against imidacloprid in an elite indica rice variety Pusa Basmati-1. Pestic. Biochem. Physiol. 105, 144–153. 10.1016/j.pestbp.2013.01.004

[B48] Sharma I. Bhardwaj R. Pati P. K. (2015). Exogenous application of 28-homobrassinolide modulates the dynamics of salt and pesticides induced stress responses in an elite rice variety Pusa Basmati-1. J. Plant Growth Regul. 34, 509–518. 10.1007/s00344-015-9486-9

[B49] Siddiqui Z. S. Ahmed S. (2006). Combined effects of pesticide on growth and nutritive composition of soybean plants. Pak. J. Bot. 38, 721–733.

[B50] Singh H. Singh N. B. Singh A. Hussain I. Yadav V. (2016). Physiological and biochemical effects of salicylic acid on *Pisum sativum* exposed to isoproturon. Arch. Agron. Soil Sci. 62, 1425–1436. 10.1080/03650340.2016.1144926

[B51] Singleton V. L. Rossi J. A. (1965). Colorimetry of total phenolics with phosphomolybdic-phosphotungstic acid reagents. Am. J. Enol. Vitic. 16, 144–158.

[B52] Tan W. Li Q. Zhai H. (2012). Photosynthesis and growth responses of grapevine to acetochlor and fluoroglycofen. Pesti. Biochem. Physiol. 103, 210–218. 10.1016/j.pestbp.2012.05.010

[B53] Tanaka K. Asami T. Yoshida S. Nakamura Y. Matsuo T. Okamoto S. (2005). Brassinosteroid homeostasis in *Arabidopsis* is ensured by feedback expressions of multiple genes involved in its metabolism. Plant Physiol. 138, 1117–1125. 10.1104/pp.104.058040 15908602 PMC1150425

[B54] Timpa J. D. Burke J. J. Quisenberry J. E. Wendt C. W. (1986). Effects of water stress on the organic acid and carbohydrate compositions of cotton plants. Plant Physiol. 82, 724–728. 10.1104/pp.82.3.724 16665100 PMC1056197

[B55] Vardhini B. V. Anjum N. A. (2015). Brassinosteroids make plant life easier under abiotic stresses mainly by modulating major components of antioxidant defense system. Front. Environ. Sci. 2:67 10.3389/fenvs.2014.00067

[B56] Wang Z. Y. Nakano T. Gendron J. He J. Chen M. Vafeados D.. (2002). Nuclearlocalized BZR1 mediates brassinosteroid-induced growth and feedback suppression of brassinosteroid biosynthesis. Dev. Cell 2, 505–513. 10.1016/S1534-5807(02)00153-3 11970900

[B57] Xi Z. M. Zhang Z. W. Huo S. S. Luan L. Y. Gao X. Ma L. N.. (2013). Regulating the secondary metabolism in grape berry using exogenous 24-epibrassinolide for enhanced phenolics content and antioxidant capacity. Food Chem. 141, 3056–3065. 10.1016/j.foodchem.2013.05.137 23871059

[B58] Xia X. J. Huang Y. Y. Wang L. Huang L. F. Yu Y. L. Zhou Y. H. (2006). Pesticides-induced depression of photosynthesis was alleviated by 24-epibrassinolide pretreatment in *Cucumis sativus* L. Pestic. Biochem. Physiol. 86, 42–48. 10.1016/j.pestbp.2006.01.005

[B59] Xia X. J. Zhang Y. Wu J. X. Wang J. T. Zhou Y. H. Shi K.. (2009). Brassinosteroids promote metabolism of pesticides in cucumber. J. Agric. Food Chem. 57, 8406–8413. 10.1021/jf901915a 19694443

[B60] Xie L. Yang C. Wang X. (2011). Brassinosteroids can regulate cellulose biosynthesis by controlling the expression of CESA genes in *Arabidopsis*. J. Exp. Bot. 62, 495–506. 10.1093/jxb/err164 21617247 PMC3170551

[B61] Ye H. Li L. Yin Y. (2011). Recent advances in the regulation of brassinosteroid signaling and biosynthesis pathways. J. Integr. Plant Biol. 53, 455–468. 10.1111/j.1744-7909.2011.01046.x 21554539

[B62] Yin Y. Vafeados D. Tao Y. Yoshida S. Asami T. Chory J. (2005). A new class of transcription factors mediates brassinosteroid-regulated gene expression in Arabidopsis. Cell 120, 249–259. 10.1016/j.cell.2004.11.044 15680330

[B63] Yuan L. B. Peng Z. H. Zhi T. T. Zho Z. Liu Y. Zhu Q. (2015). Brassinosteroid enhances cytokinin-induced anthocyanin biosynthesis in *Arabidopsis* seedlings. Biol. Plant. 59, 99–105. 10.1007/s10535-014-0472-z

[B64] Zhou X. Y. Song L. Xue H. W. (2013). brassinosteroids regulate the differential growth of *Arabidopsis* hypocotyls through auxin signaling components IAA19 and ARF7. Mol. Plant 6, 887–904. 10.1093/mp/sss123 23125315

[B65] Zhou Y. Xia X. Yu G. Wang J. Wu J. Wang M.. (2015). Brassinosteroids play a critical role in the regulation of pesticide metabolism in crop plants. Sci Rep. 5:9018. 10.1038/srep09018 25761674 PMC4356967

[B66] Zhu W. Wang H. Fujioka S. Zhou T. Tian H. Tian W. (2013). Homeostasis of brassinosteroids regulated by DRL1, a putative acyltransferase in Arabidopsis. Mol. Plant 6, 546–558. 10.1093/mp/sss144 23204503

